# Establishing a density-based method to separate proliferating and senescent cells from bone marrow stromal cells

**DOI:** 10.18632/aging.103569

**Published:** 2020-07-25

**Authors:** Fei Xu, Qiang Zhang, Haitao Wang

**Affiliations:** 1 Department of Orthopedics, Tongji Hospital, Tongji Medical College, Huazhong University of Science and Technology, Wuhan, China; 2 Department of Orthopaedics, Shanghai East Hospital, Tongji University School of Medicine, Shanghai, China; 3 Department of Physiology Biomedical Engineering, Division of Geriatric Medicine and Gerontology, and the Robert and Arlene Kogod Center on Aging, Mayo Clinic, Rochester, MN 55905, USA

**Keywords:** bone marrow stromal cells, cell transplantation, density-based method, proliferating cells, senescent cells

## Abstract

To assist in the study of cellular aging, we established a new method of enriching physiologically aged bone marrow stromal cells (BMSCs) in animals of any age using a Percoll density gradient centrifugation technique. BMSCs from mice 2 months of age were isolated, and their cellular age determined (over 80% Scal-1^+^ CD29^+^ CD11b^-^ CD45^-^ CD105^-^ and able to differentiate into osteoblasts, adipocytes, and chondrocytes). As proof –of principle, cells were aged in vitro and confirmed by low bromodeoxyuridine (BrdU) incorporation and senescence-associated β-galactosidase (SA-β-gal) staining. Proliferating cells were enriched in high-density gradient layers, and senescent cells were enriched in low-density gradient layers. We confirmed that over 80% of cells from the low-density gradient layer were senescent with SA-β-gal staining and telomere dysfunction-induced foci (TIF) assay. This density-based method, which can separate proliferating and senescent BMSCs, could be used to study mechanisms of physiologic cell aging and may have implications for the use of BMSCs in clinical transplant applications.

## INTRODUCTION

Bone marrow stromal cells (BMSCs) are adherent, express the surface markers of stem cells, and preserve the capacity to differentiate into osteocytes, adipocytes, and chondrocytes. BMSCs are essential cell sources for studying the aging process and for application in clinical bone marrow transplantation. A substantial limitation in studying the aging process is the shortage of an efficient method to enrich the low number of senescent cells from naturally occurring senescence. Alternatively, senescent cells are generated by treatment with radiation, chemical agents, or derived from genetic models of accelerated aging [1, 2]. However, none of these approaches fully repeat physiologic aging at the cellular level.

On the other hand, BMSCs are widely used in Crohn disease, bone fracture, acute and chronic graft-versus-host disease, acute myocardial infarction, cerebrospinal injury, and cerebrovascular diseases. The intrinsic heterogeneity of BMSCs limits their clinical application. A small number of senescent cells can be detrimental to the outcome of mesenchymal stem cell transplant [3]. The functional study of senescent cells is further limited by the lack of a method to effectively separate proliferating and senescent cells.

It is essential to establish a method to separate proliferating and senescent cells. However, the scarcity of robust senescent markers and reduced cell viability after isolation limit the clinical application of traditional isolation methods (eg, magnetic bead isolation, flow cytometry) [4, 5]. A method of ultrahigh throughput isolation and removal of senescent cells with chips based on cell size increase during cellular senescence has recently been reported [6].

Morphologic features of senescent cells usually include enlargement, flattening, vacuolization, and, occasionally, multinucleation [7]. The lower nucleus to cytoplasm ratio of senescent cells—which yields a lower specific density than proliferating cells—could be used to aid in the separation of senescent and proliferating cells in mixed primary or other cultures. Okumura et al [8] and Kovacovicova and Vinciguerra [9] established a density-gradient centrifugation method of eliminating senescent cells to purify cultured corneal endothelial cells for cell therapy. However, no separation method exists for BMSCs. Here, we propose a simple procedure for separating senescent and proliferating BMSCs from cultured BMSCs by density-gradient centrifugation.

## RESULTS

### Isolation and identification of BMSCs

**Figure 1:**
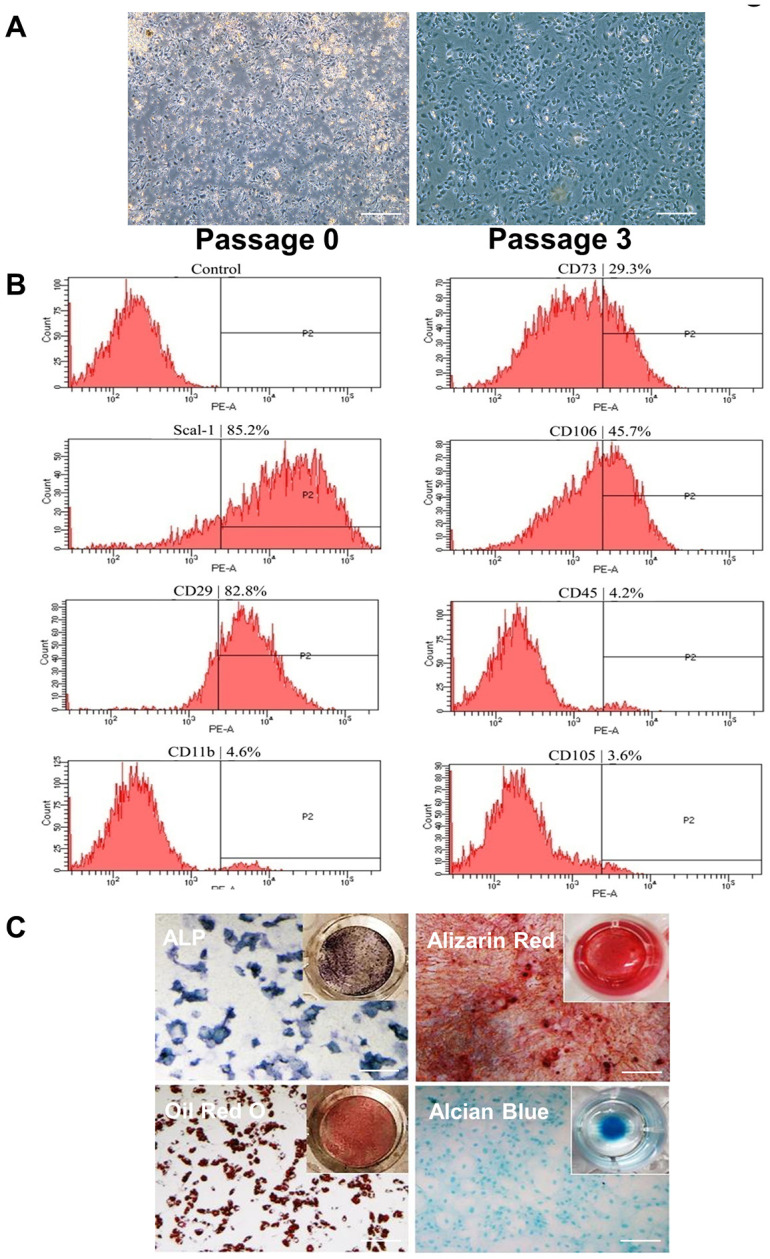
**Isolation and identification of BMSCs by phenotypic characterization and multipotent differentiation potential.** (**A**) Cells were isolated from the femurs and tibias of 3- to 4-week-old mice shown at P0 and P3. Cells are attached at P3. Scale Bar=200μm. (**B**) Flow cytometric analysis of cell surface markers on isolated BMSCs indicates Scal-1^+^ CD29^+^ CD11b^-^ CD45^-^ CD105^-^. (**C**) Differentiation capacity of BMSCs: ALP staining of cells cultured in osteogenic induction medium for 7 days (upper-left image); alizarin red staining of cells cultured in osteogenic induction medium for 21 days (upper-right image); oil red O staining of cells cultured in adipogenic induction medium for 7 days (lower-left image); and alcian blue staining of cells cultured in chondrogenic induction medium for 14 days (lower-right image). P0, passage 0; P2, passage 2; P3, passage 3. Scale Bar=200μm.

BMSCs were analyzed and identified by surface markers at passage 3 (P3) using flow cytometry. As shown in Figure 1A, all cells are adherent to the flask wall; in Figure 1B, BMSCs express cell surface proteins Scal-1^+^ CD29^+^ CD11b^-^ CD45^-^ CD105^-^; and in Figure 1C, the isolated cells reserve trilineage differentiation capacity. Osteogenic differentiation ability was shown using alkaline phosphatase (ALP) staining of cells cultured in osteogenic induction medium for 7 days (upper-left image) and alizarin red staining of cells cultured in osteogenic induction medium for 21 days (upper-right image). Adipogenic differentiation ability was shown using oil red O staining of cells cultured in induction medium for 7 days (lower-left image). Chondrogenic induction was shown using alcian blue staining of cells cultured inchondrogenic medium for 14 days (lower-right image).

### Separation of proliferating and senescent cells by density-gradient centrifugation

**Figure 2:**
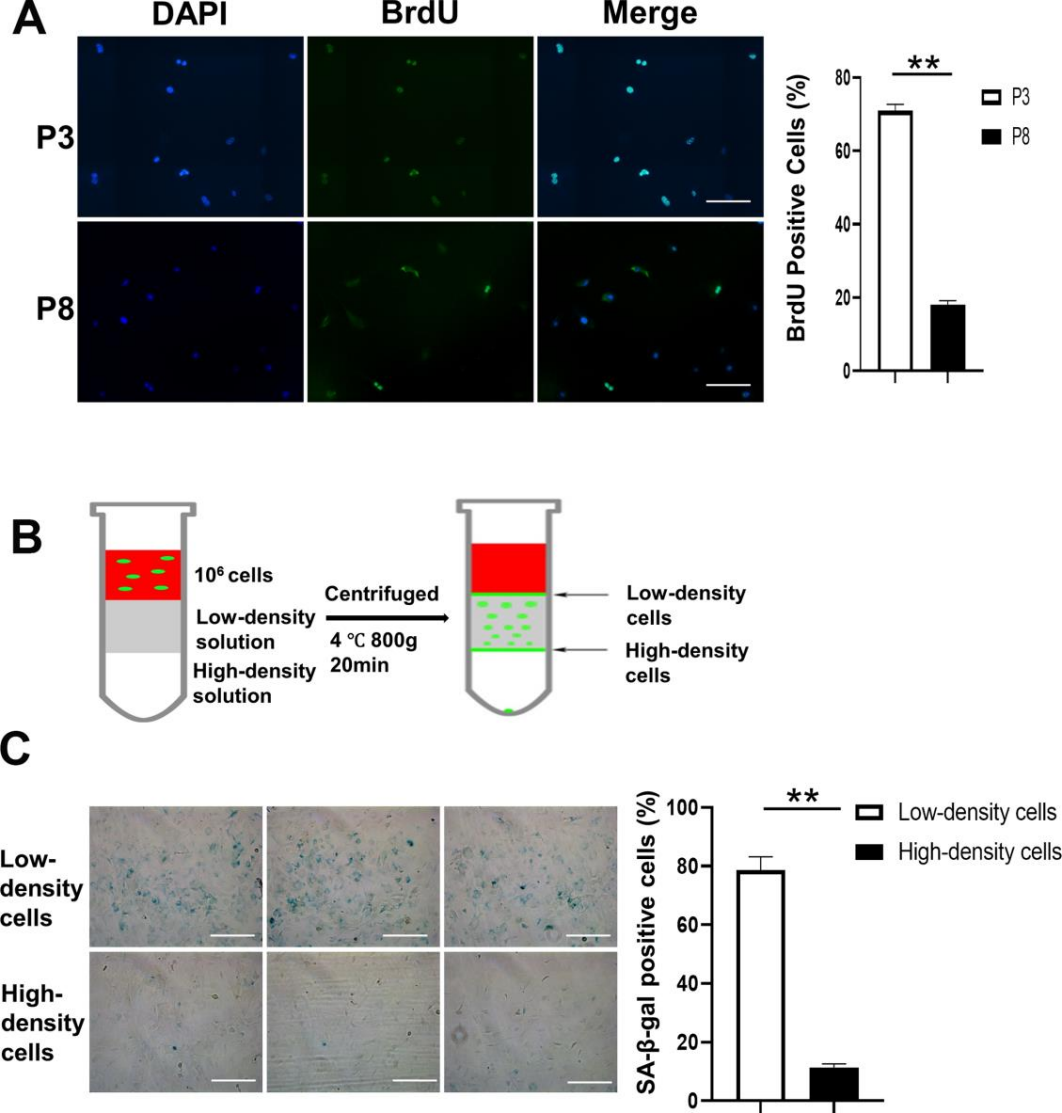
**Density gradient separation of proliferating and senescent cells.** (**A**) Immunofluorescence of BrdU positive cells is shown in the first panel. The right panel shows that the number of BrdU positive cells is significantly lower in P8 compared to P3. Scale Bar=200μm. (**B**) BMSCs (P8), a mixture of proliferating and senescent cells, were centrifuged through a density gradient medium (OptiPrep, Sigma-Aldrich) at 800g for 20 minutes. Aliquots (0.5 mL) were taken from the low- and high-density layers. The cells were then incubated in a 48-well plate. (**C**) SA-β-gal staining of the 2 groups. Low-density cells contained a higher percentage of SA-β-gal positive cells compared to the high-density cells. The right panel shows that the number of SA-β-gal positive cells is significantly lower in high-density cells compared to low-density cells (n=3). BrdU indicates bromodeoxyuridine; DAPI, 4′,6-diamidino-2-phenylindole; P3, passage 3; P8, passage 8; SA-β-gal, senescence-associated β-galactosidase; Paired T-Test **, *P*<.01. data are represented as mean ± SEM. Scale Bar=200μm.

**Figure 3:**
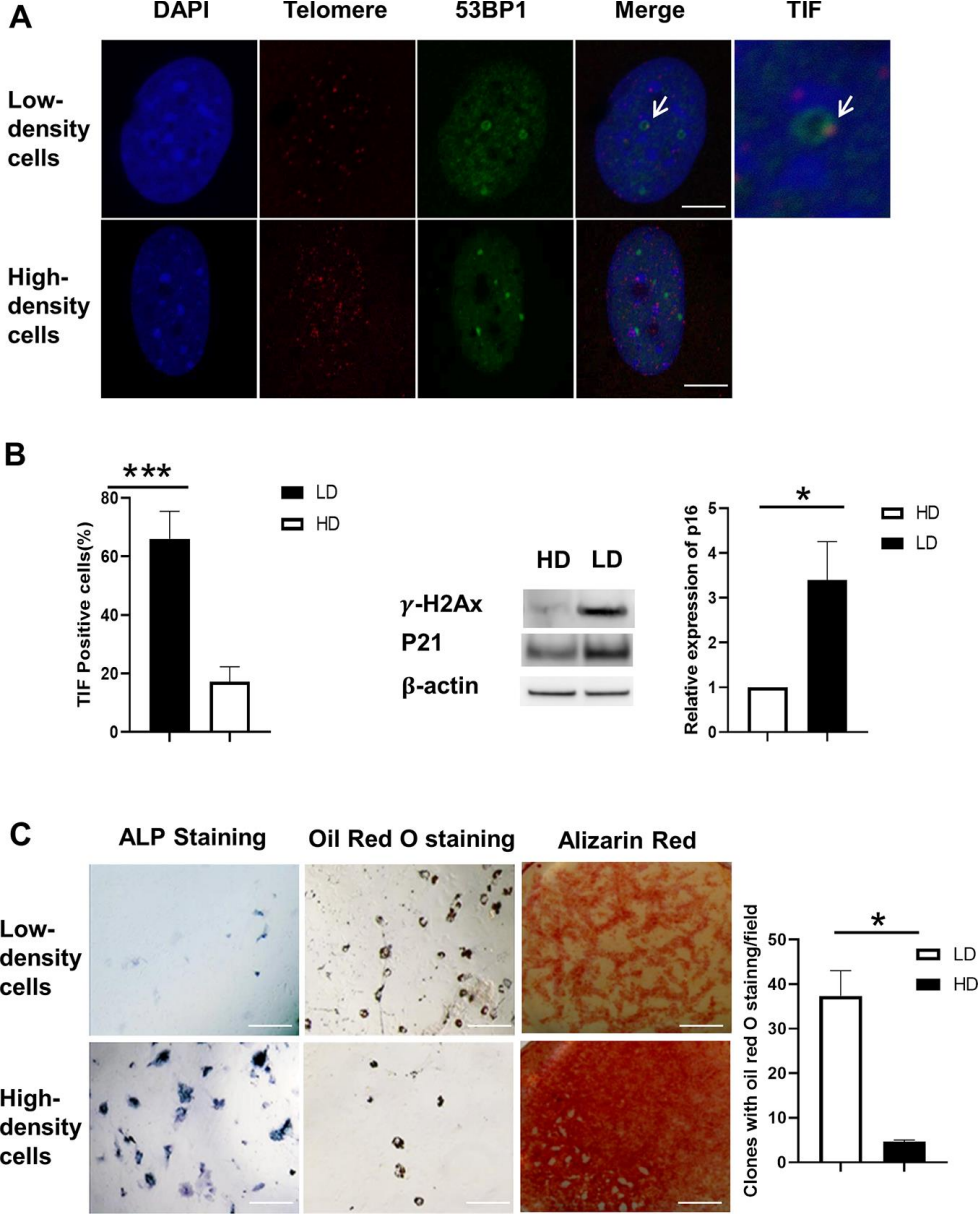
**Differentiation capacity and TIF assay of BMSCs after separation.** (**A**) Co-localization of 53BP1 DNA damage protein (green) and telomeric DNA (red). TIFs are indicated by arrows. Blue, DAPI; Red, Telomere; Orange/Yellow, TIFs. Scale Bar=5μm. (**B**) The percentage of TIF-positive cells is substantially lower in high-density cells compared to low-density cells (left panel). The protein levels of γH2AX and P21 were induced at low density (LD) using Western blot. *P16* gene expression was increased at LD using real-time PCR. (**C**) Low- and high-density cells were cultured either in osteogenic induction medium or adipogenic induction medium for 7 days. ALP and oil red O staining were conducted at the end of 14 days. ALP staining (upper- and lower-left panels) was induced in the high-density cell group. Oil red O staining (upper- and lower-middle panels) was induced in the low-density cell group. Alizarin red staining (upper- and lower-right panels) was induced in the high-density cell group. Quantification of clones of oil red O staining positive (n=3) is shown in the graph at right. ALP indicates alkaline phosphatase; DAPI, 4′,6-diamidino-2-phenylindole; HD, high density; LD, low density; TIF, telomere dysfunction-induced foci. Paired T-Test, ***, *P*<.001, data are represented as mean ± SEM. Scale Bar=200μm.

BMSCs were isolated and cultured from passage 0 (P0) to passage 8 (P8). Cell proliferation was detected by bromodeoxyuridine (BrdU) assay. As shown in Figure 2A, more BrdU-positive BMSCs appear at P3 than at P8, where they are mixed with proliferating and senescent cells. Figure 2B shows the density-gradient separation system we used to separate proliferating and senescent cells at P8. The cells from the 2 groups were collected, and senescence-associated β-galactosidase (SA-β-gal) staining was conducted. A higher percentage of SA-β-gal positive cells was found in the low-density cells than in the high-density cells. Also, detection of TIFs showed 66% of TIF-positive cells in the low-density cell group compared to 17.2% in the high-density cell group. Protein levels of γH2AX and P21 were induced in cells from the low-density layer using Western blot assay, and *P16* gene expression was increased at the low-density layer using real-time polymerase chain reaction (PCR) (Figure 3A and 3B).

### Differentiation capacity of BMSCs in separated proliferating and senescent cells

Low- and high-density cells were cultured either in osteogenic induction medium or adipogenic induction medium for 7 days. ALP and oil red O staining were conducted at the end of the indicated time. ALP staining in the low-density cell group showed more high-density cells, and oil red O staining in the high-density cell group showed more low-density cells (Figure 3C).

## DISCUSSION

In this project, we established a density-gradient based method to isolate proliferating cells and enrich the senescent cells from BMSCs through continual culturing *in vitro*. We found that the lower-layer cells are proliferating cells with low TIFs and SA-β-gal staining, and they differentiate well into multiple lineage cells (eg, osteogenic, chondrogenic, adipogenesis). The higher-layer cells are enriched senescent cells with high TIFs on SA-β-gal staining and do not adequately differentiate into multiple lineage cells.

For research purposes, the isolated senescent cells from naturally aged mice by a novel method provide an excellent cell source for studying the mechanism of cellular aging. The enriched senescent cells isolated from middle-aged mice or from continually culturing cells in vitro may be a valuable source to identify early molecular events of aging.

For clinical transplant applications, the isolated proliferating cells also provide an optimization method for cell transplant therapy. The effect of BMSC-based therapy is limited due to poor engraftment and limited regenerative potential. The efficacy of BMSC transplantation in clinical settings can be affected by various traits, including donor cell factors (eg, safety, autologous or allogeneic, *ex vivo* cell expansion), patient factors (eg, age, stroke type), treatment factors (eg, interval since onset, delivery route, cell dose), and validation factors (eg, neurologic assessment, imaging) [10–12]. Among these factors, aging is associated with decreased maximal life span and accelerated senescence of BMSCs. The separated proliferating BMSCs may provide a source for better engraftment and regenerative potential.

Our study has limitations. We could not get pure cell populations after centrifuge. This may be attributable to the heterogeneous sources of BMSCs, which were mixed with hematopoietic lineage cells, even though we used the cells after P3, there were still other cell types mixed in. Furthermore, the complexity of senescent cells makes it difficult to separate them from proliferating cells with a method based solely on cell density. Other methods (eg, cell sorting with magnetic beads based on molecular markers) could be combined with the density-based method to enrich more pure cell populations.

In summary, we provide a novel density-gradient based method to isolate proliferating cells and enrich the senescent cells from BMSCs from naturally aged mice. The separated proliferating cells could be applied for BMSC transplant, and the senescent cells may be a suitable cell source to study the mechanisms of aging. The density-gradient based method could also be combined with other separating methods to enrich more specific cell types.

## MATERIALS AND METHODS

### Isolation and identification of BMSCs from mice

Three- to 4-week-old mice were euthanized by CO_2_ inhalation, and the femurs and tibiae of the mice collected for BMSC isolation. BMSCs were harvested by flushing with α-minimal essential medium (α-MEM) (Thermo Fisher Scientific) containing 10% fetal bovine serum (FBS) (Thermo Fisher Scientific) and penicillin/streptomycin (100 U/mL/100 μg/mL) (Thermo Fisher Scientific). The cells were cultured in a T25 flask at 37 °C in a 5% CO_2_ humidified incubator. The nonadherent cells were removed after 72 hours, and the adherent cells were cultured continually. When the cells reached 90% confluence, 0.05% trypsin-ethylenediaminetetraacetic acid was used to split the cells. The medium was changed every 3 days. The cells from P3 were used to test phenotypic characterization and multipotent differentiation potentials.

### Flow cytometric analysis

Mouse mesenchymal stem cell marker antibody panel (Research And Diagnostic Systems, Inc) was used to identify and characterize the P3 cells. Briefly, the cells were suspended in flow cytometry staining buffer at a concentration of 1×10^6^ cells/mL, and different antibodies were added for each marker, including anti-Scal-1^+^, anti-CD29^+^, anti-CD11b^-^, anti-CD45^-^, and anti-CD105. After a 30-minute incubation period, samples were centrifuged and washed in flow cytometry staining buffer. The second antibodies were then added to the sample and incubated for 30 minutes in the dark. Following incubation, centrifugation, and sample washing, the cells were suspended for flow cytometric analysis. All live cells were counted, and the percentage of positive cells were analyzed individually with different markers.

### Differentiation capacity of mouse BMSCs

P3 BMSCs were used to test osteoblast, adipocyte, and chondrocyte differentiation capacity. To determine osteoblast-induced activity, BMSCs were cultured in osteogenesis medium (α-MEM, 10% FBS, 10 nM dexamethasone [Sigma-Aldrich Corp], 50 μmol/L ascorbic acid [Sigma-Aldrich Corp], and 10 mM β-glycerophosphate [Sigma-Aldrich Corp]). The differential medium was changed every 3 days. After 7 days of culture, the samples were stained with a specific ALP substrate 5-bromo-4-chloro-3-indolyl-phosphate (Moss, Inc) in the presence of p-nitro blue tetrazolium chloride to detect ALP activity staining. After 21 days of culture, the samples were stained by alizarin red S to detect calcium nodes and observed under a microscope. For adipocyte induction, BMSCs were cultured in adipogenesis medium (α-MEM, 10% FBS, 0.1 mM 3-isobutyl-1-methylxanthine, 10 mg/L insulin, 0.1 mM indomethacin, and 1 μM dexamethasone). The medium was changed every 3 days. After 7 days of culture, samples were stained by oil red O to detect lipid droplets. To determine chondrocyte-induced activity, BMSCs were cultured in chondrogenesis medium (α-MEM, 10% FBS, 10 ng/mL Bone Morphogenetic Protein 2 [Research and Diagnostics Systems, Inc], 100 nM dexamethasone [Sigma-Aldrich Corp], 50 μg/mL ascorbic acid 2-phosphate [Sigma-Aldrich Corp], 1 mM sodium pyruvate [Sigma-Aldrich Corp], 40 μg/mL proline [Sigma-Aldrich Corp], and 1X insulin-transferrin-selenium [Thermo Fisher Scientific]). After 21 days of culture, samples were stained by alcian blue and observed under a microscope.

The same osteogenesis medium, adipogenesis medium, and detection protocol were used to evaluate the ability of differentiating proliferating cells and senescent cells that were separated. After induction, the cells were stained with an ALP staining kit (Moss, Inc) and oil red O and alizarin red S staining to examine the ability of differentiation to osteoblast and adipocyte of proliferating cells and senescent cells.

### Separation of proliferating cells and senescent cells by density-gradient centrifugation

The proliferation of BMSCs at P3 and BMSCs at P8 was tested by BrdU staining. Briefly, the cells were plated 24 hours in advance and incubated with 20 μM BrdU (Sigma-Aldrich Corp). The cells were washed with 1X Phosphate-buffered saline (PBS) twice and fixed by 4% formaldehyde, then permeabilized with Triton X-100. After washing, the cells were incubated with 1N hydrogen chloride and incubated with a 1:500 dilution of anti-BrdU antibody (Thermo Fisher Scientific) overnight. The cells were washed with PBS and incubated with secondary antibody at room temperature for 3 hours, then we mounted cells with Vecta-shield with 4′,6-diamidino-2-phenylindole (DAPI).

A density gradient medium (OptiPrep, Sigma-Aldrich) was used to separate proliferating cells and senescent cells. The separate density of gradient medium was 1.058 g/ml prepared, per product protocol. The separation system is shown in Figure 2B. The BMSCs at P8 were suspended in α-MEM at a density of 1×10^6^ cells/mL. For the separated system, the upper layer was 2 mL cell solution, the mid layer was 2 mL 1.058 g/ml gradient solution, and the lower layer was 2 mL 1.222 g/ml gradient solution (the density is much higher than the density of the cells). The cells were then centrifuged at 800 g for 20 minutes. The cells from the upper level and lower level were collected separately and incubated in a 6-well plate or 48-well plates.

### SA-β-gal staining

Cells in the 48-well plates were stained with SA-β-gal by Senescence β-Galactosidase Staining Kit (Cell Signaling Technology) in accordance with product instructions. The percentage of positive cells were counted and shown (Figure 2C).

### TIF assay

Paraformaldehyde-fixed BMSCs were stained by telomere TIF assay, as described previously [1]. At least 100 nuclei were examined for each sample, and the number of TIFs (telomere and 53BP1 co-localized foci) was counted. TIF-positive cells were defined as those with at least 1 TIF foci. Confocal analysis was performed at Mayo Clinic on a Zeiss LSM510 META NLO laser scanning confocal microscope using a Plan-Apo 63x/1.4 oil objective. Image capture was performed using Zeiss LSM510 META version 4.2 software.

### Western blot analysis

Total cell protein was dissolved in RIPA buffer containing 150 mM NaCl, 1.0% IGEPAL CA-630, 0.5% sodium deoxycholate, 0.1% SDS, and 50 mM Tris, pH 8.0 (Sigma-Aldrich Corp) with 1× proteinase inhibitor cocktail (Sigma-Aldrich Corp) and phosphatase inhibitor cocktail (Pierce). The lysate was incubated on ice for 15 minutes and centrifuged at 12,000 g for 10 minutes. The amount of protein in the supernatant was quantified using the BCA Protein Assay kit (Pierce). Fifty μg of total protein was separated on a 10% SDS-PAGE gel and then transferred to PVDF membranes (Bio-Rad Laboratories) by electroblotting. Membranes were incubated at 4 °C overnight with a 1:1,000 dilution of antibody specific for γH2AX (Novus Biologicals), a 1:500 dilution of antibody specific for P21 (Santa Cruz Biotechnology, Inc), and a 1:1,000 dilution of antibody specific for β-actin antibody (Santa Cruz Biotechnology, Inc).

### Real-time PCR

Cells were washed with PBS, and RNA was isolated using the RNeasy kit (QIAGEN) following the manufacturer's instructions. cDNA was synthesized with the Super-Script VILO cDNA Synthesis Kit (Life Technologies). Real-time PCR reactions containing forward and reverse primers, cDNA (1:5 dilution), and SYBR Green PCR Master Mix (Applied Biosystems) were performed by standard methods. Each sample was analyzed in triplicate with the 7500 Sequence Detection System (Applied Biosystems), and target gene mRNA levels were quantified from threshold cycles (ΔΔCT method) and normalized to 18S rRNA. The following primer sequences were used: GAPDH 5′-TGAAGGTCGGTGTGAACGGATTTGGC-3′ (forward), 5′-CATGATGGCCATGAGGTCCACCAC-3′ (reverse), *P16*^ink4a^ 5’-GAACTCTTTCGGTCGTACCC-3’ (forward), 5’-AGTTCGAATCTGCACCGTAGT -3’ (reverse).

### Statistical analysis

The data are presented as SEM. The 2-sample comparison was determined with the 2-tailed paired *t* test. *P* values less than .05 were considered statistically significant.
